# Supplementary material for: Shaping lightwaves in time and frequency for optical fiber communication
Source: Nat Commun. 2022 Feb 10;13:785. doi: 10.1038/s41467-022-28349-x (PMC8831651; doi:10.1038/s41467-022-28349-x)
Supplement: Supplementary file 1 — Supplementary Information [file 41467_2022_28349_MOESM1_ESM.pdf]

Supplementary Figures

The supplementary figures provided herein show extensive results of the split-step and EGN model simulations, which support the analysis of the influence of sphere shaping on Kerr nonlinearity presented in the article.

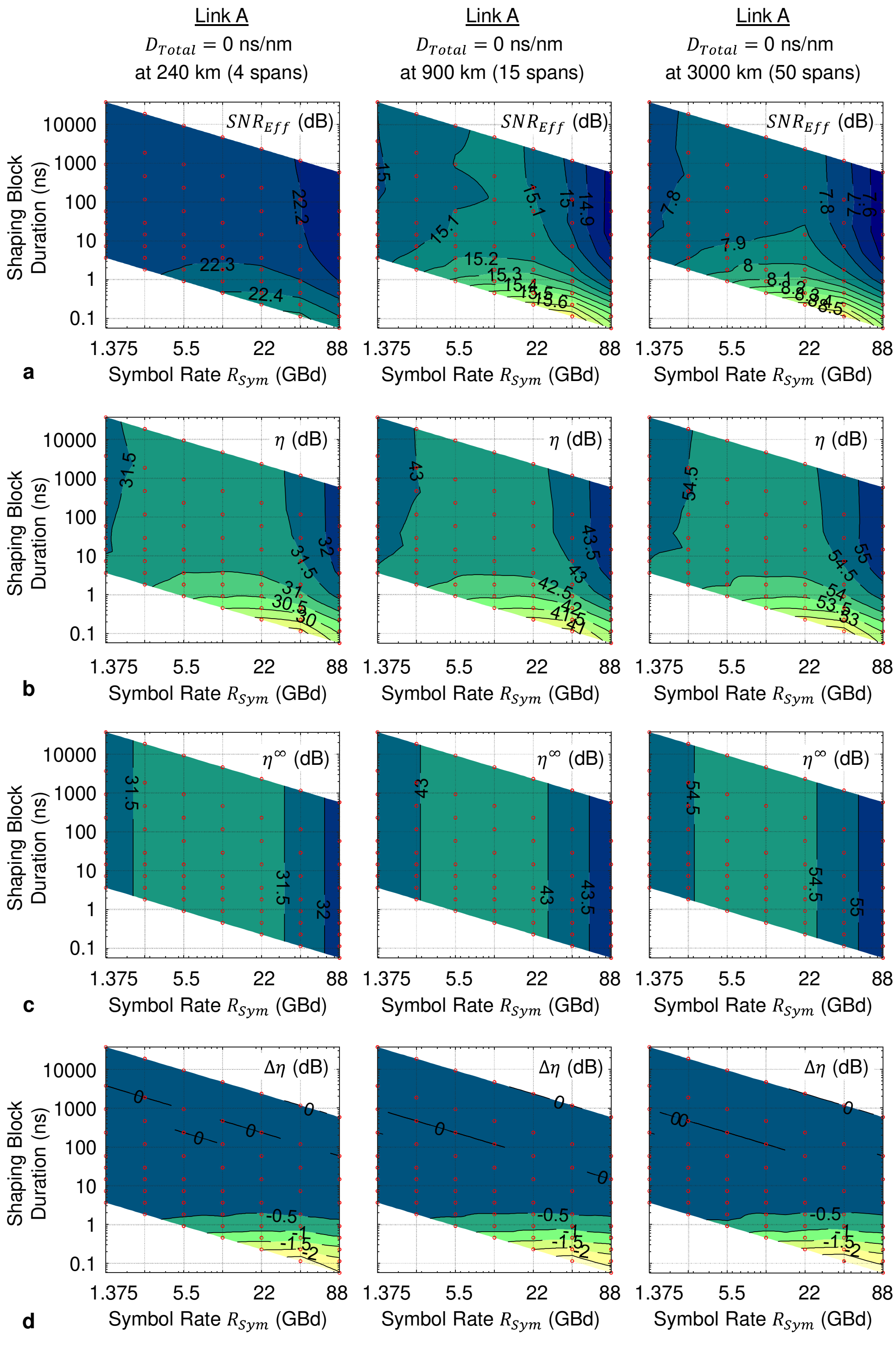

Supplementary Figure 1 Split-step simulation results for Link A at 240 km (left figures), 900 km (middle figures), and 3000 km (right figures). **a** Effective SNR (dB) drawn as a function of the symbol rate and shaping block duration. **b** NLI coefficient (dB). **c** NLI coefficient for near-i.i.d. shaped symbols (dB). **d** Change of NLI coefficient due to finite-length shaping.

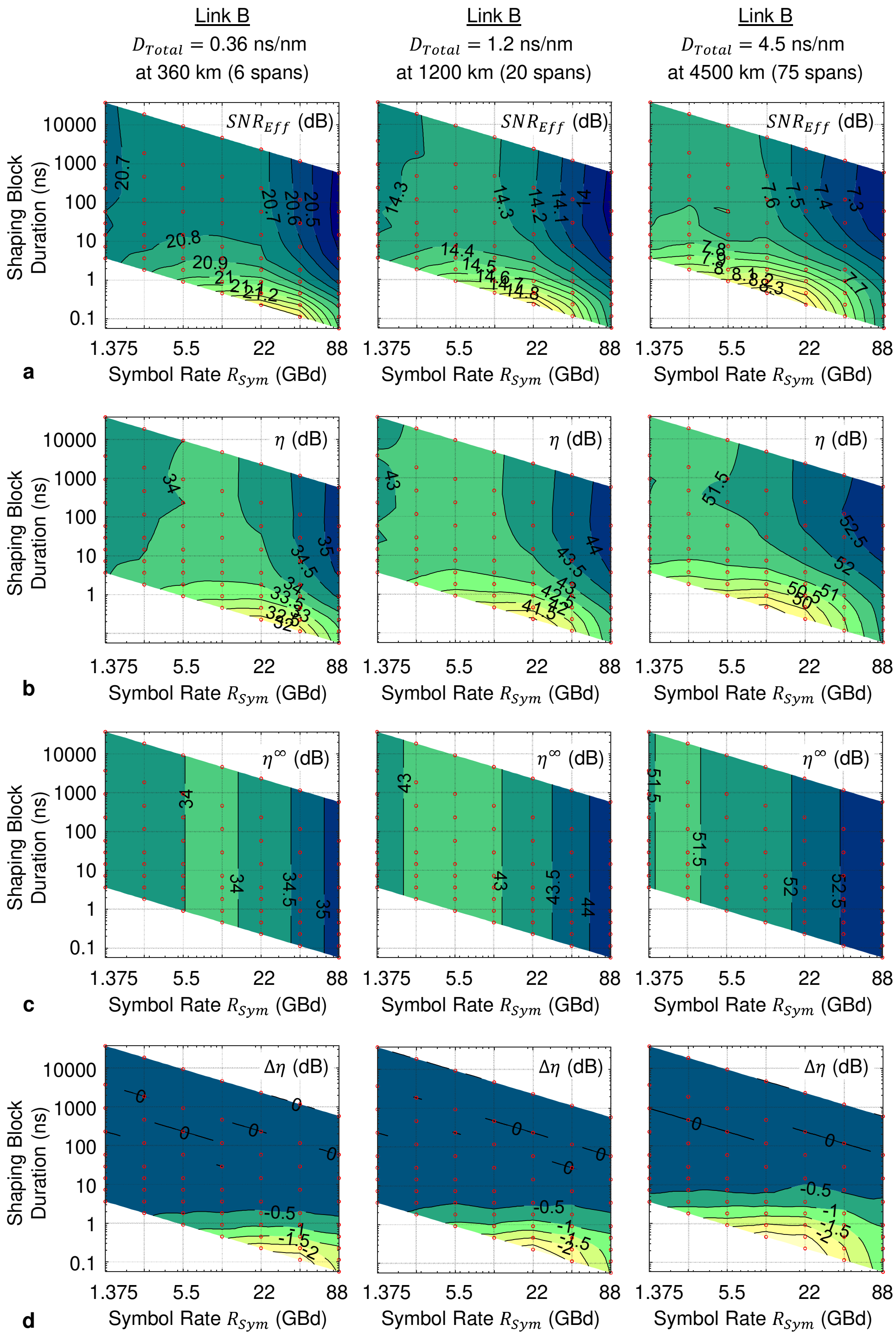

Supplementary Figure 2 Split-step simulation results for Link B at 360 km (left figures), 1200 km (middle figures), and 4500 km (right figures). a Effective SNR (dB) drawn as a function of the symbol rate and shaping block duration. **b** NLI coefficient (dB). **c** NLI coefficient for near-i.i.d. shaped symbols (dB). **d** Change of NLI coefficient due to finite-length shaping.

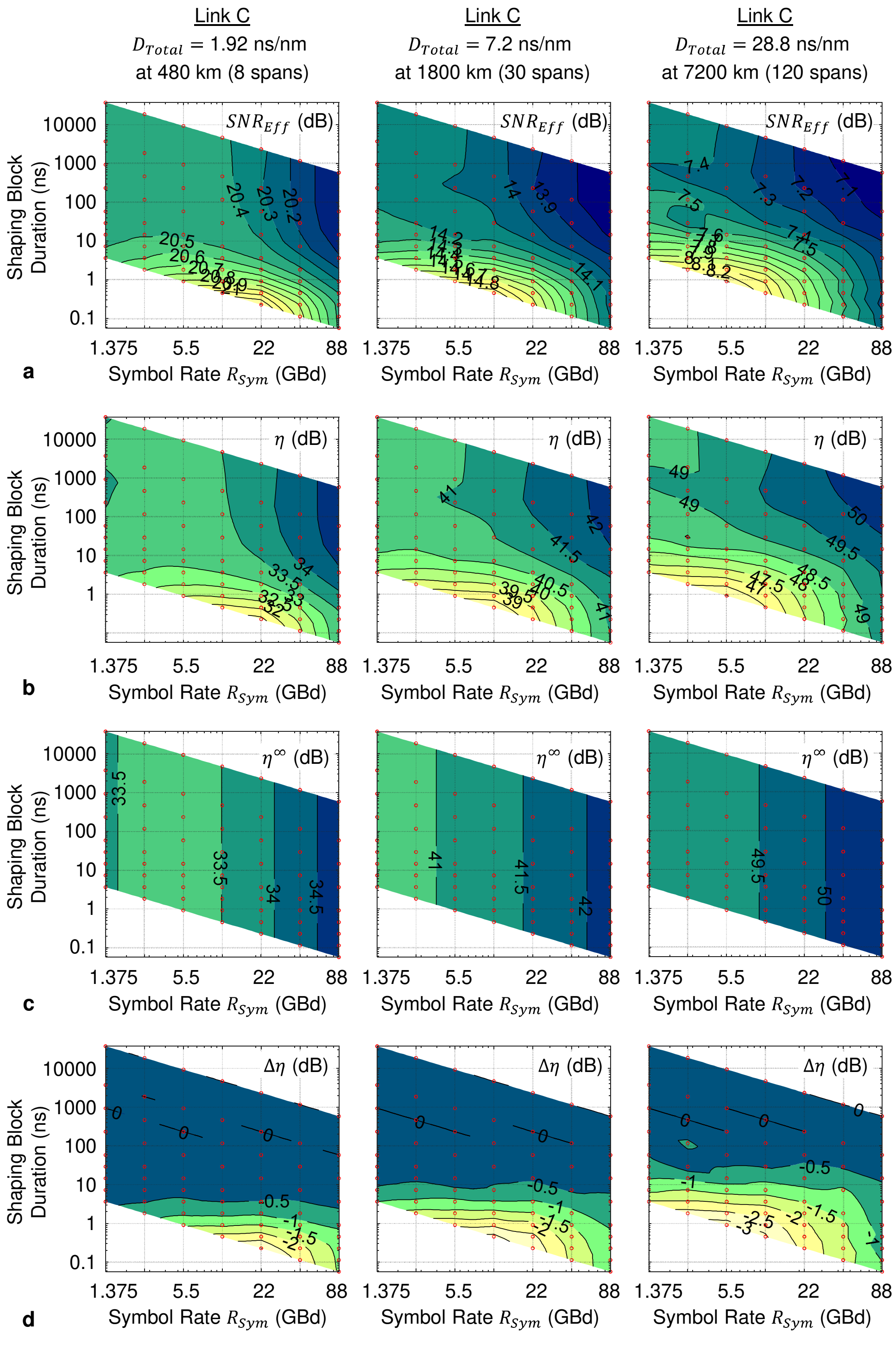

Supplementary Figure 3 Split-step simulation results for Link C at 480 km (left figures), 1800 km (middle figures), and 7200 km (right figures). **a** Effective SNR (dB) drawn as a function of the symbol rate and shaping block duration. **b** NLI coefficient (dB). **c** NLI coefficient for near-i.i.d. shaped symbols (dB). **d** Change of NLI coefficient due to finite-length shaping.

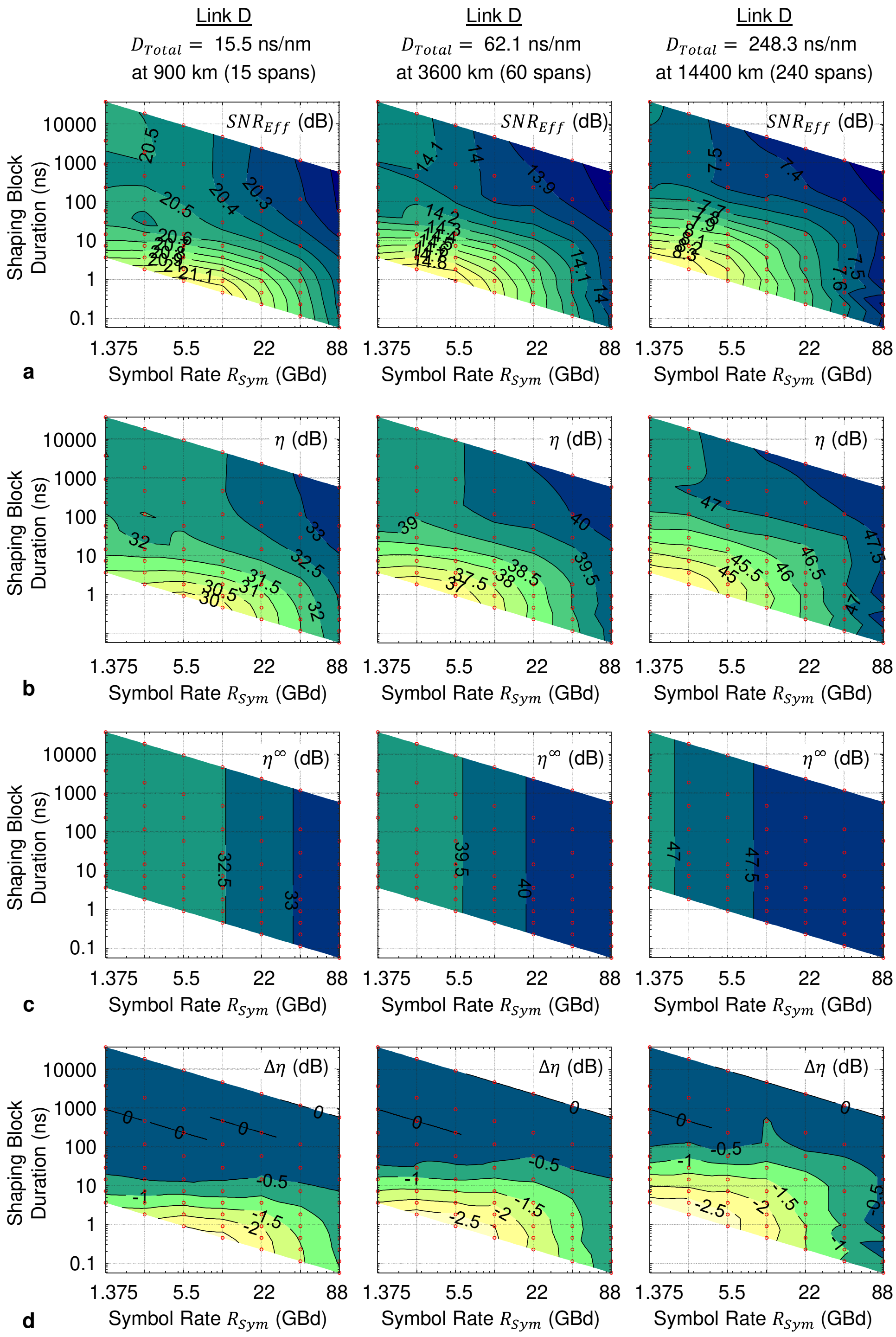

Supplementary Figure 4 Split-step simulation results for Link D at 900 km (left figures), 3600 km (middle figures), and 14400 km (right figures). a Effective SNR (dB) drawn as a function of the symbol rate and shaping block duration. **b** NLI coefficient (dB). **c** NLI coefficient for near-i.i.d. shaped symbols (dB). **d** Change of NLI coefficient due to finite-length shaping.

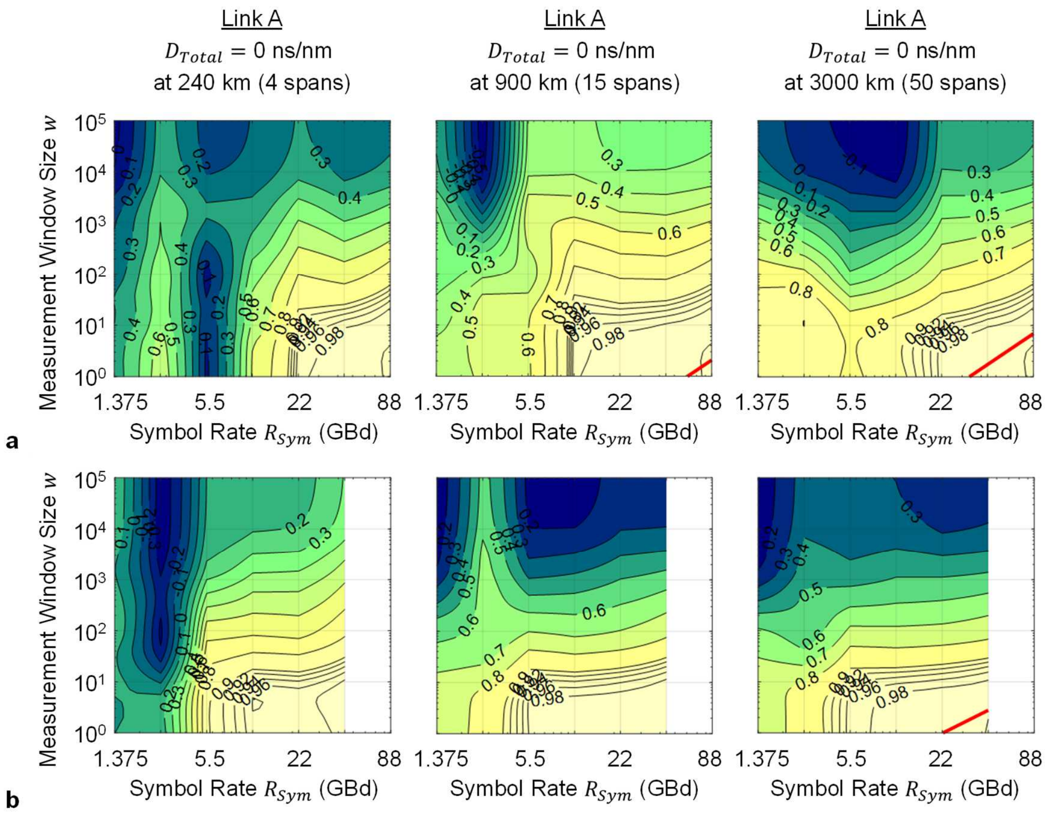

Supplementary Figure 5 Pearson correlation coefficient obtained in Link A at 240 km (left figures), 900 km (middle figures), and 3000 km (right figures). a Correlation between η_{SPM} and $\bar{\mu}_2$. **b** Correlation between η_{XPM} and $\bar{\mu}_2$. In (**b**), there is no XPM at $R_{Sym} = 88$ GBd, since only one channel is transmitted. The red lines in (**a**) and (**b**) are obtained with equations (7) and (8), which are estimated to produce the greatest correlations.

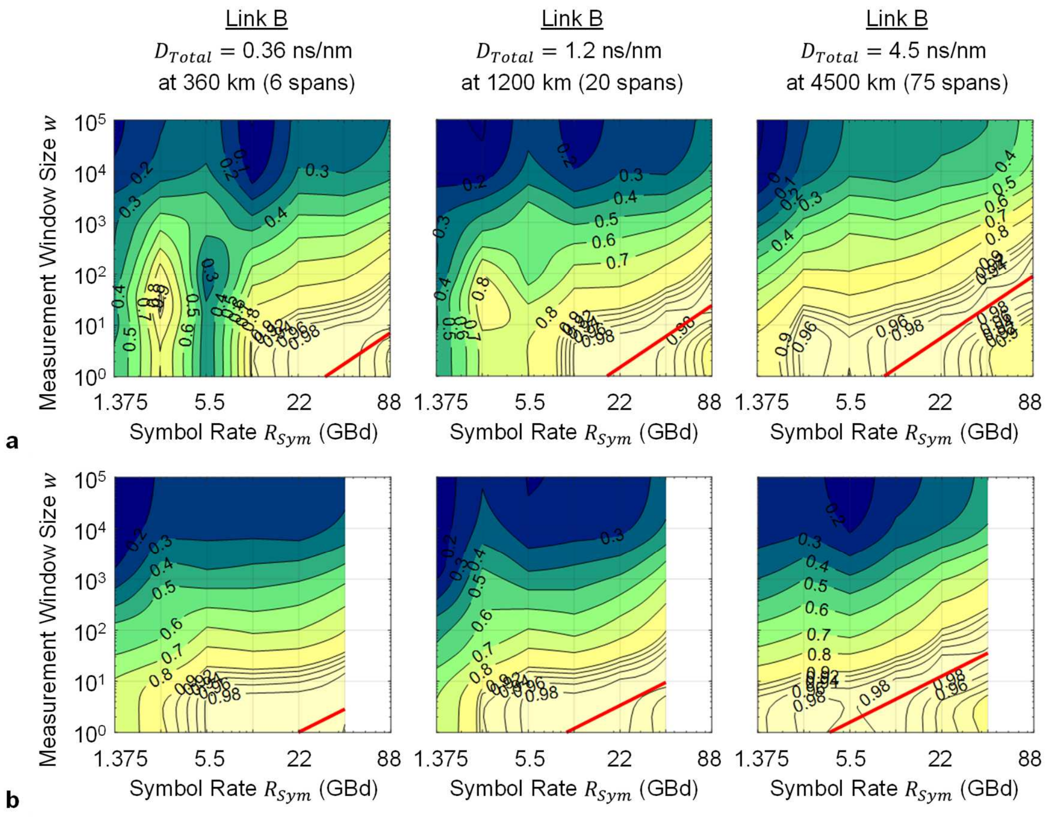

Supplementary Figure 6 Pearson correlation coefficient obtained in Link B at 360 km (left figures), 1200 km (middle figures), and 4500 km (right figures). a Correlation between η_{SPM} and $\bar{\mu}_2$. **b** Correlation between η_{XPM} and $\bar{\mu}_2$. In (**b**), there is no XPM at $R_{Sym} = 88$ GBd, since only one channel is transmitted. The red lines in (**a**) and (**b**) are obtained with equations (7) and (8), which are estimated to produce the greatest correlations.

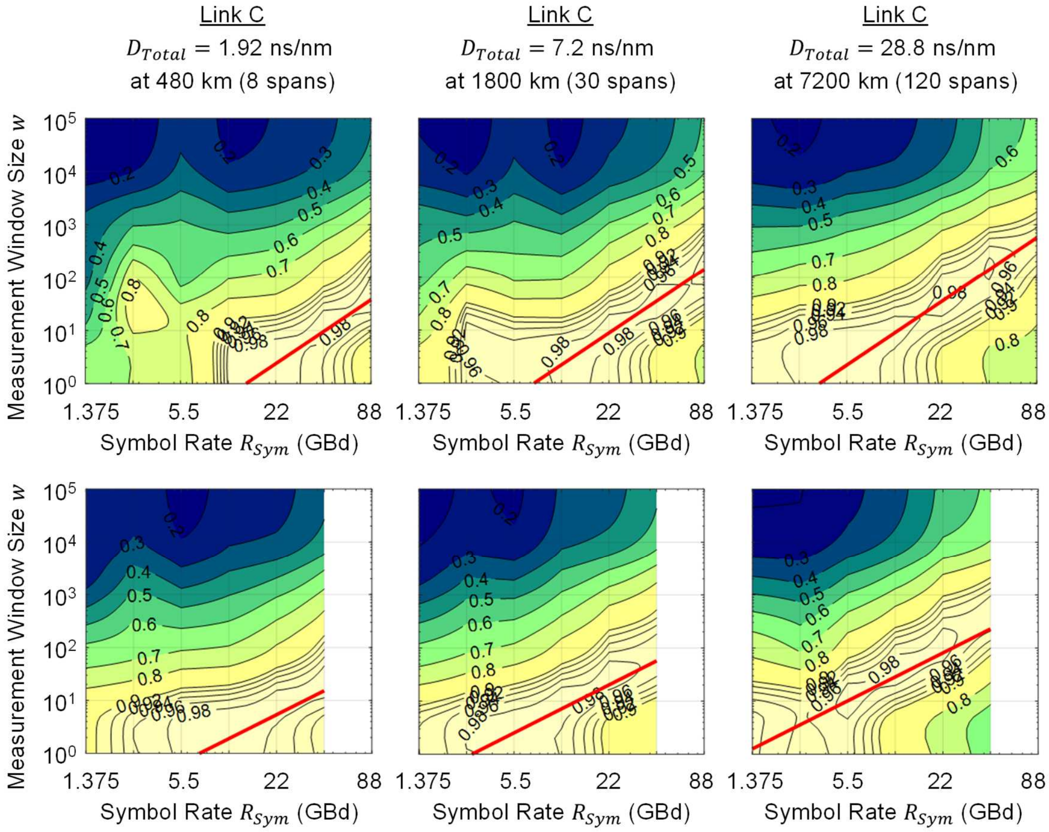

Supplementary Figure 7 Pearson correlation coefficient obtained in Link C at 480 km (left figures), 1800 km (middle figures), and 7200 km (right figures). a Correlation between η_{SPM} and $\bar{\mu}_2$. **b** Correlation between η_{XPM} and $\bar{\mu}_2$. In (**b**), there is no XPM at R_{Sym} = 88 GBd, since only one channel is transmitted. The red lines in (**a**) and (**b**) are obtained with equations (7) and (8), which are estimated to produce the greatest correlations.

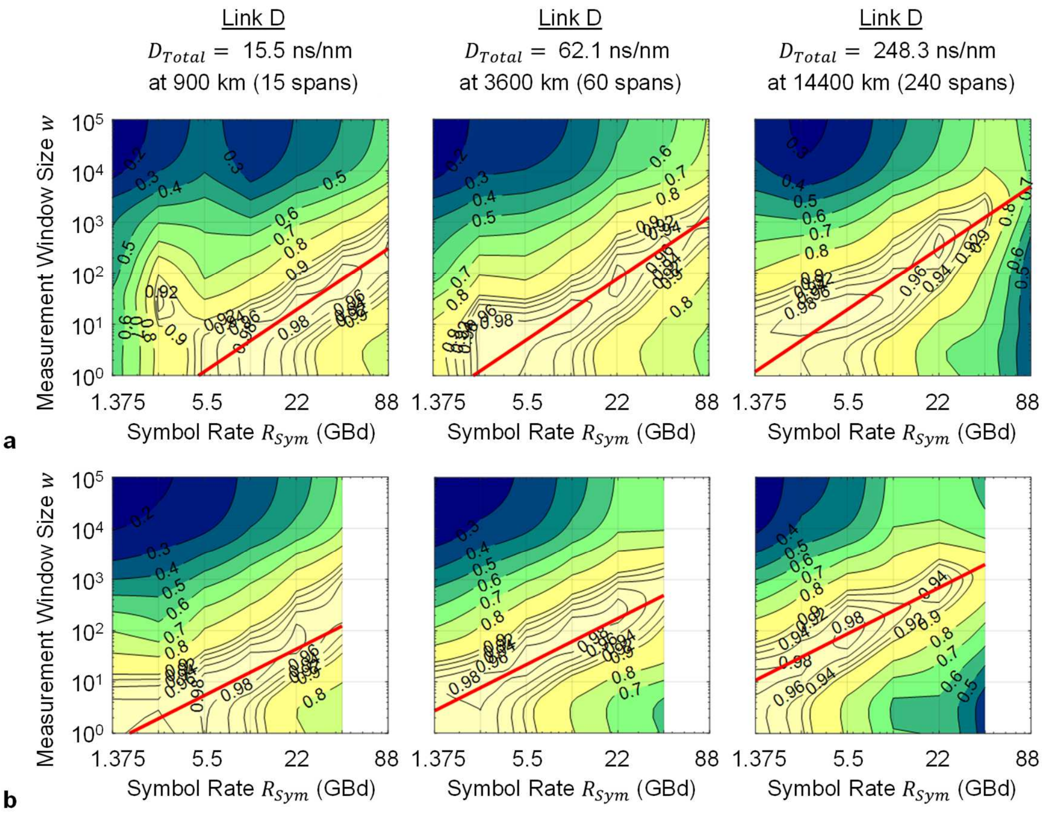

Supplementary Figure 8 Pearson correlation coefficient obtained in Link D at 900 km (left figures), 3600 km (middle figures), and 14400 km (right figures). a Correlation between η_{SPM} and $\bar{\mu}_2$. **b** Correlation between η_{XPM} and $\bar{\mu}_2$. In (**b**), there is no XPM at $R_{Sym} = 88$ GBd, since only one channel is transmitted. The red lines in (**a**) and (**b**) are obtained with equations (7) and (8), which are estimated to produce the greatest correlations.

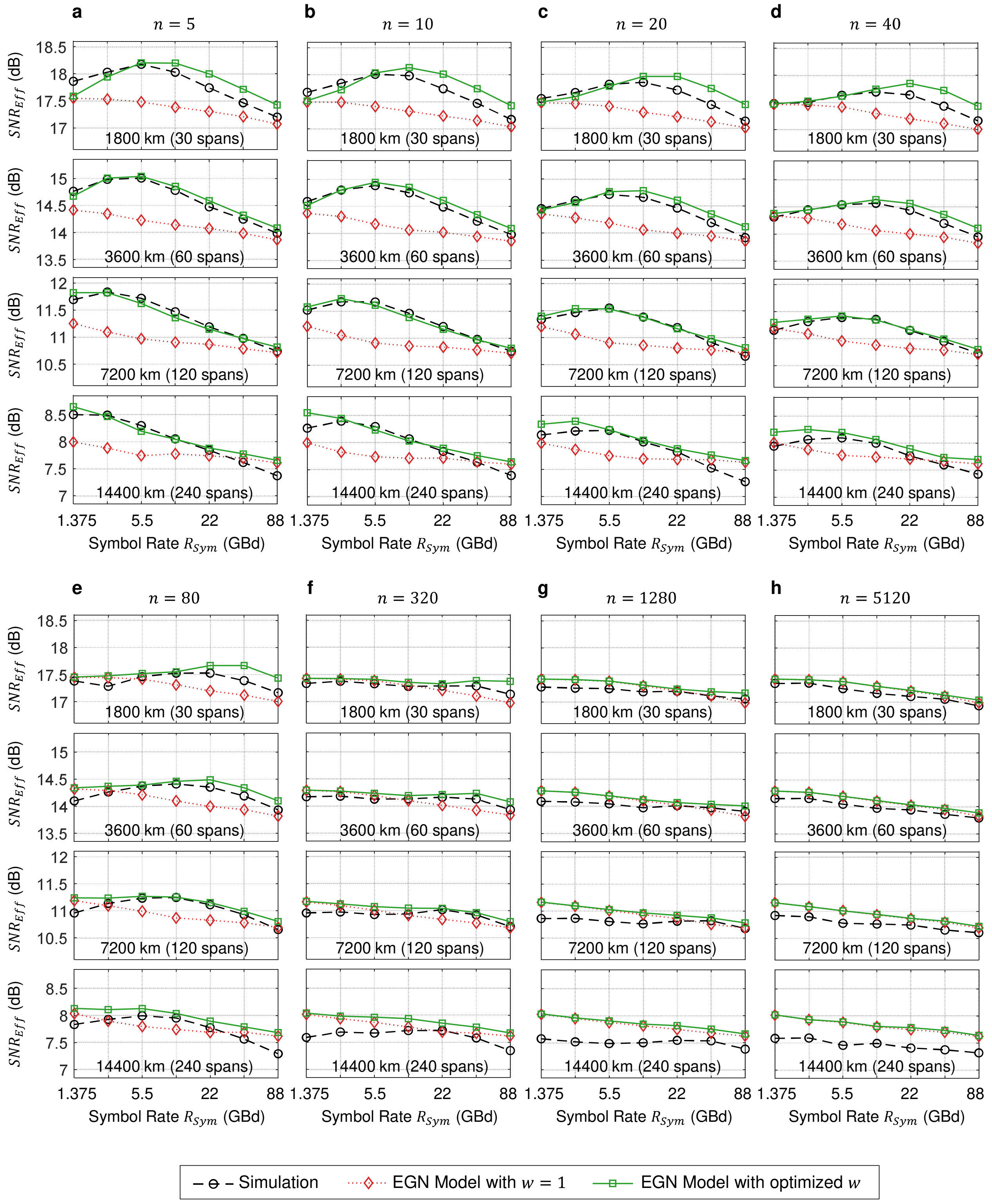

Supplementary Figure 9 Comparison of the results of split-step simulation and EGN model simulation with and without windowing the instantaneous power measurement, in Link D. a $n = 5$. **b** $n = 10$. **c** $n = 20$. **d** $n = 40$. **e** $n = 80$. **f** $n = 320$. **g** $n = 1280$. **h** $n = 5120$.

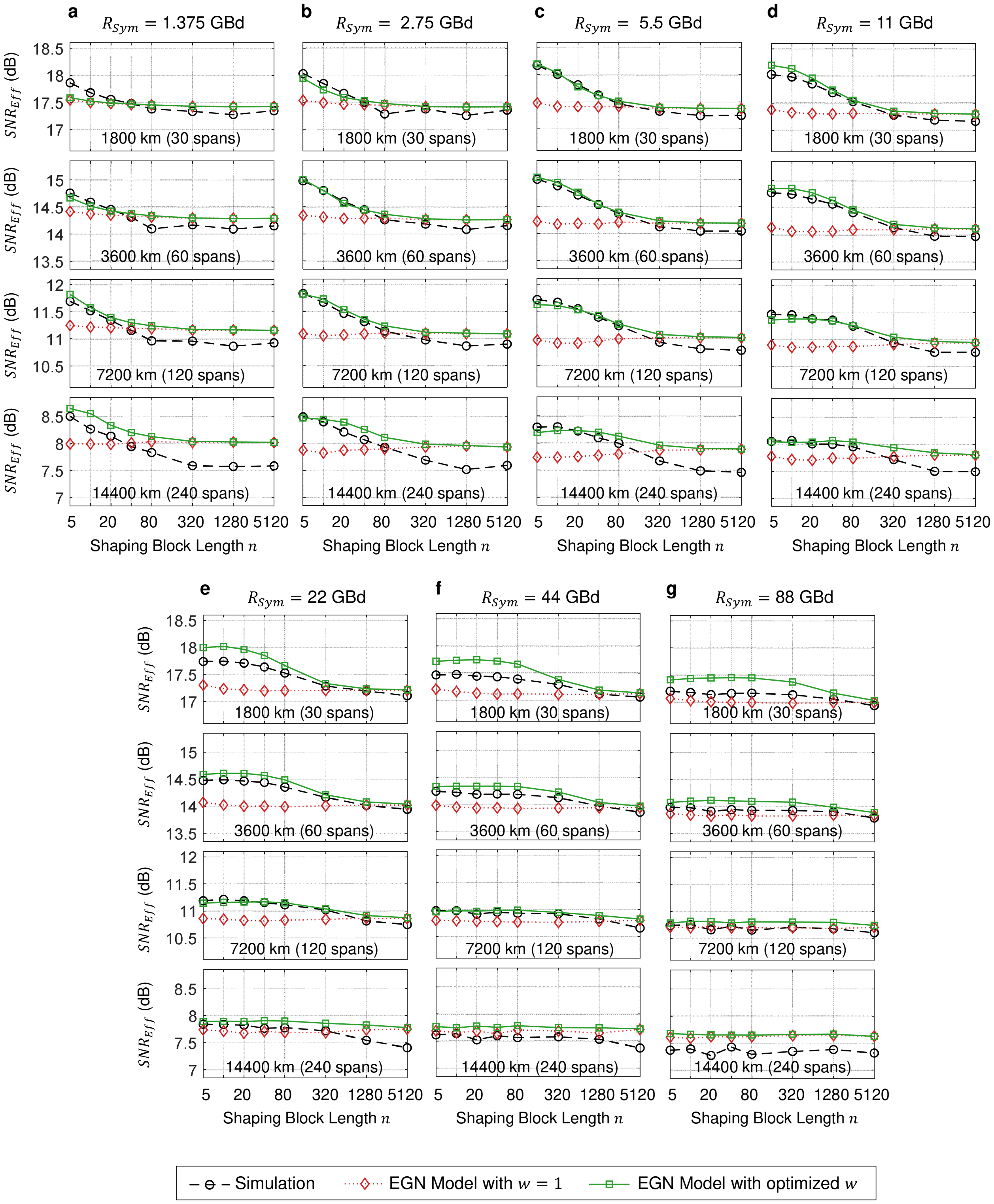

Supplementary Figure 10 Comparison of the results of split-step simulation and EGN model simulation with and without windowing the instantaneous power measurement, in Link D. a R_{Sym} = 1.375 GBd. **b** R_{Sym} = 2.75 GBd. **c** R_{Sym} = 5.5 GBd. **d** R_{Sym} = 11 GBd. **e** R_{Sym} = 22 GBd. **f** R_{Sym} = 44 GBd. **g** R_{Sym} = 88 GBd.
